# Interplay of Kinetic and Thermodynamic Factors in the Stationary Composition of Vapor–Liquid–Solid IIIV_x_V_1−x_ Nanowires

**DOI:** 10.3390/nano14161333

**Published:** 2024-08-09

**Authors:** Vladimir G. Dubrovskii, Egor D. Leshchenko

**Affiliations:** 1 Faculty of Physics, St. Petersburg State University, Universitetskaya Emb. 13B, 199034 St. Petersburg, Russia; 2 Submicron Heterostructures for Microelectronics, Research and Engineering Center RAS, Politekhnicheskaya Street, 26, 194021 St. Petersburg, Russia; leschenko.spbu@mail.ru

**Keywords:** III–V ternary nanowires, group V intermix, VLS growth, composition, modeling

## Abstract

Compositional control over vapor–liquid–solid III–V ternary nanowires based on group V intermix (VLS IIIV_x_V_1−x_ NWs) is complicated by the presence of a catalyst droplet with extremely low and hence undetectable concentrations of group V atoms. The liquid–solid and vapor–solid distributions of IIIV_x_V_1−x_ NWs at a given temperature are influenced by the kinetic parameters (supersaturation and diffusion coefficients in liquid, V/III flux ratio in vapor), temperature and thermodynamic constants. We analyze the interplay of the kinetic and thermodynamic factors influencing the compositions of VLS IIIV_x_V_1−x_ NWs and derive a new vapor–solid distribution that contains only one parameter of liquid, the ratio of the diffusion coefficients of dissimilar group V atoms. The unknown concentrations of group V atoms in liquid have no influence on the NW composition at high enough levels of supersaturation in liquid. The simple analytic shape of this vapor–solid distribution is regulated by the total V/III flux ratio in vapor. Calculating the temperature-dependent desorption rates, we show that the purely kinetic regime of the liquid–solid growth occurs for VLS IIIV_x_V_1−x_ NWs in a wide range of conditions. The model fits the data well on the vapor–solid distributions of VLS InP_x_As_1−x_ and GaP_x_As_1−x_ NWs and can be used for understanding and controlling the compositions of any VLS IIIV_x_V_1−x_ NWs, as well as modeling the compositional profiles across NW heterostructures in different material systems.

## 1. Introduction

Due to a very small footprint in contact with lattice-mismatched substrates and in axial NW heterostructures, freestanding III–V ternary NWs and NW-based heterostructures offer almost unlimited possibilities for bandgap engineering and the integration of novel optoelectronic devices with the Si electronic platform [1,2,3,4,5,6,7,8]. Most III–V ternary NWs are grown by the VLS method [9,10,11] with a metal catalyst, often Au [9], which is replaced by a group III metal in the self-catalyzed VLS approach [10]. III–V ternary NWs can be based on a group V (IIIV_x_V_1−x_ NWs) [12,13,14,15,16,17,18,19,20,21,22,23,24,25,26,27,28,29,30,31,32,33] or group III (III_x_III_1−x_V NWs) [34,35,36,37] intermix. Compositional control over VLS IIIV_x_V_1−x_ NWs [12,13,14,15,16,17,18,19,20,21,22] and heterostructures within such NWs [4,5,23,24,25,26,27,28,29,30,31,32,33] by tuning the growth parameters (temperature, vapor composition and total V/III flux ratio) and compositional modeling [36,37,38,39,40,41,42,43,44,45,46] is much more complex than for VLS III_x_III_1−x_V NWs [34,35,36,37]. This is mainly explained by (i) extremely low concentrations of highly volatile group V elements such as As and P in liquid (~1%), which are below the detection limit of any characterization techniques, and (ii) the high desorption rates of these elements from a catalyst droplet [39,40]. The latter are extremely sensitive to temperature and droplet composition [40]. Group III atoms are more stable at the typical VLS growth temperatures. As a result, their loss by desorption or “negative” diffusion from a catalyst droplet is almost negligible. In this case, the vapor–solid distribution x(z), connecting the solid composition in an A_x_B_1−x_C NW with the fraction of group III A atoms in vapor z, is simply given by the one-parameter Langmuir–McLean (LM) formula [47]. The sole parameter of this distribution, $c_{g}$, describes different diffusivities of A and B adatoms on the surfaces surrounding an NW. The LM shape describes quite well the measured vapor–solid distributions of most Al_x_Ga_1−x_As, In_x_Ga_1−x_As and In_x_Ga_1−x_N NWs, without any miscibility gaps in the last two material systems [46]. This is generally not the case for VLS IIIV_x_V_1−x_ NWs, for which even the identification of the control parameters influencing their composition remains challenging [40,41]. Consequently, the required level of theoretical understanding and compositional control of VLS IIIV_x_V_1−x_ NWs in the technologically important material systems is solely lacking.

In this work, we address the problem by developing the approach of Refs. [39,40,41] for modeling the stationary compositions of VLS IIIV_x_V_1−x_ NWs. In these works, we have obtained the kinetic liquid–solid distribution of VLS A_x_B_1−x_C NWs, connecting the NW composition with the fraction of group V A atoms in liquid. This kinetic form is guaranteed for any liquid catalyst, because the liquid–solid growth of the ternary NW monolayer (ML) always proceeds under group III-rich conditions [39]. This property is simple and fundamental for understanding the compositional trends in VLS III–V ternary NWs. Furthermore, we have shown that the uncertainty of the liquid phase (including the unknown group V concentrations and the related interactions in liquid) can be circumvented in many important cases [40,41]. For example, the approximate vapor–solid distribution obtained in Ref. [40] explains the data of Ref. [12], where the x(z) dependence of Au-catalyzed InSb_x_As_1−x_ NWs transitioned from the linear curve x=z at low total V/III flux ratios to a markedly non-linear shape at high V/III ratios. This effect was earlier reported and modeled for InSb_x_As_1−x_ epi-layers [48]. However, the influence of the liquid state on the stationary vapor–solid distribution of VLS IIIV_x_V_1−x_ NWs should depend on the material system and growth conditions. Consequently, the interplay of kinetic and thermodynamic factors at a given temperature and total V/III requires in-depth investigation.

Here, we study this complex interplay for different IIIV_x_V_1−x_ NWs and apply our findings to modeling the available experimental data for VLS IIIPAs NWs. We find that the vapor–solid distribution of VLS IIIP_x_As_1−x_ NWs can be reduced to a simple analytic shape given by a combination of the linear curve x=z and a non-linear term with a single parameter. The latter contains the ratio of the pre-factors in the desorption rates of B_2_ over A_2_ dimers times the squared ratio of their diffusion coefficients in pure group V liquids ${\left(D_{A}/D_{B}\right)}^{2}$. Calculations of the desorption rates using the thermodynamic data for P_2_, As_2_ and Sb_2_ dimers over a wide range of temperatures show that the vapor–solid distribution is independent of the liquid composition in nearly all cases, apart from exotic growth conditions (such as very high temperatures ~600 °C for GaSbAs NWs). With the known desorption rates, fitting the available data on the compositions of VLS InP_x_As_1−x_ and GaP_x_As_1−x_ NWs allows us to deduce the ratios $D_{A}/D_{B}$ for a set of temperatures. This was not accessed before to our knowledge. For the first time, our analysis shows that the stationary vapor–solid distributions of VLS IIIV_x_V_1−x_ in a wide range of growth conditions depend only on temperature and the effective total V/III ratio, where all the temperature-dependent functions are lumped together in a single control parameter. These results should be useful for the compositional control over VLS IIIV_x_V_1−x_ NWs and are essential for modeling the interfacial abruptness across VLS NW heterostructures based on group V interchange. They are also required for the time-dependent generalizations of the model accounting for the depletion of a catalyst with its group V atoms with the ML progression.

## 2. Model

Consider the stationary VLS growth of an A_x_B_1−x_C NW based on group V interchange, with A and B atoms belonging to group V and C atoms belonging to group III. The liquid composition is defined as the fraction of A atoms in liquid:

(1) $$y=\frac{\chi_{A}}{\chi_{A}+\chi_{B}}.$$

Here, $\chi_{A}$ and $\chi_{B}$ are the atomic concentrations of group V atoms A and B in liquid, with $\chi_{tot}=\chi_{A}+\chi_{B}$ as their total concentration. The concentration of group III C atoms in liquid equals $\chi_{C}$, with $\chi_{Au}=1-\chi_{C}-\chi_{tot}$ as the concentration of Au atoms in the Au-catalyzed VLS growth and $\chi_{Au}=0$ in the self-catalyzed VLS growth. Group V atoms incorporate from liquid to solid in the excess of group III atoms. According to Refs. [39,40], this feature leads to the liquid–solid distribution of VLS IIIV_x_V_1−x_ NWs of the following kinetic form [45]:

$$y=\frac{x+g(x)}{x+c_{l}(1-x)},\;g\left(x\right)=\Gamma_{l}x\left(1-x\right)\left[c_{l}e^{\omega {\left(1-x\right)}^{2}}-\beta_{l}e^{\omega x^{2}}\right],$$

$$c_{l}=\frac{D_{A}}{D_{B}}e^{\psi_{A}-\psi_{B}},$$

$$\beta_{l}=e^{∆\mu_{AC}^{0}-∆\mu_{BC}^{0}+\psi_{A}-\psi_{B}},$$

(2) $$\Gamma_{l}=\frac{1}{{\chi_{tot}\chi }_{C}}e^{-∆\mu_{AC}^{0}-\psi_{A}-\psi_{C}}.$$

Here, ω is the pseudo-binary interaction parameter of the AC and BC pairs in solid in thermal units. This parameter increases with the lattice mismatch between AC and BC binary materials, and yields the miscibility gaps for III–V ternaries at low temperatures corresponding to ω>2. $D_{k}$ denotes the diffusion coefficients of k= A, B atoms. The functions $\psi_{k}$ describe the interaction terms in the chemical potentials of the group V A and B atoms in liquid, given by $\mu_{A}^{l}=\mu_{A}^{l,0}+ln\chi_{A}+\psi_{A}$, $\mu_{B}^{l}=\mu_{B}^{l,0}+ln\chi_{B}+\psi_{B}$. Here, $\mu_{k}^{l,0}$ denotes the chemical potentials of pure k= A, B and C liquids, and $\mu_{kC}^{s,0}$ denotes the chemical potentials of solid binaries AC and BC in thermal units. The quantities ${∆\mu_{AC}^{0}=\mu }_{A}^{l,0}+{\mu_{C}^{l,0}-\mu }_{AC}^{s,0}$ and ${∆\mu_{BC}^{0}=\mu }_{B}^{l,0}+{\mu_{C}^{l,0}-\mu }_{BC}^{s,0}$ in Equation (2) for $\beta_{l}$ and $\Gamma_{l}$ are the chemical potential differences for pure binaries. With neglect of small corrections, the interaction terms $\psi_{k}$ are determined by $\chi_{C}$ and are independent of y and $\chi_{tot}$. In Ref. [39], the normalized diffusion fluxes of atoms A and B in liquid were defined as $j_{k}=-D_{k}\nabla [exp(\psi_{k}{)\chi }_{k}]$, which corresponds to $j_{k}=-D_{k}\nabla \chi_{k}$ at $\psi_{k}=0$ for the pure liquids A and B. Hence, $D_{k}$ in Equation (2) for $c_{l}$ is the diffusion coefficient of atoms A and B in pure liquids A and B. With the interaction terms in Equation (2), $c_{l}$ gives the ratio of diffusion coefficients of A over B atoms in a Au-group III melt with a given fraction of Au, or in pure group III liquid without any Au in the self-catalyzed VLS growth. Overall, the liquid–solid distribution given by Equation (2) is controlled by (i) the different transport of atoms A and B through liquid (the parameter $c_{l}$), (ii) interactions in solid (the parameter ω) and (iii) the different thermodynamic stabilities of atoms A and B in liquid (the affinity parameter $\beta_{l}$). These purely thermodynamic factors are lumped together in the function g(x). The weight of thermodynamic factors is determined by the supersaturation parameter $\Gamma_{l}$. At $\;\Gamma_{l}\to 0$ (high levels of supersaturation in liquid), thermodynamics does not affect the liquid–solid distribution. A larger $\Gamma_{l}$ (lower supersaturations) leads to thermodynamically controlled compositions. This will be elaborated in more detail below.

The liquid–solid distribution given by Equation (2) is general and requires no assumptions [39]. It should be noted, however, that the total group V content in liquid $\chi_{tot}$ is so low that a catalyst droplet may get depleted with its group V atoms in the instantaneous process of ML progression. This may lead to interesting effects such at the stopping size corresponding to zero supersaturation of liquid, after which the partial ML grows at the rate of refill from vapor [49,50,51,52]. Such liquid–solid growth may also be considered using Equation (2), where both y and ${\;\chi }_{tot}$ become time-dependent and evolve differently in different ML growth steps. However, the complex growth regimes with a stopping size and generally with a considerable droplet depletion and a time-scale hierarchy in the ML progression were never studied in the compositional modeling and require a separate treatment. In this work, we assume a time-independent y and ${\;\chi }_{tot}$. This requires large enough droplets and high enough concentrations of group V atoms in liquid.

The liquid–solid distribution given by Equation (2) depends on many parameters, including the purely thermodynamic constants  ω and $\beta_{l}$, and the kinetic constants $c_{l}$ and $\Gamma_{l}$. The parameter $\Gamma_{l}$ is inversely proportional to the effective supersaturation of atoms A in liquid with respect to a binary solid AC. At $\Gamma_{l}\ll 1$, the liquid–solid distribution is reduced to the purely kinetic LM shape [40].

(3) $$y_{kin}=\frac{x}{x+c_{l}(1-x)}.$$

In this regime, the incoming fluxes of atoms A and B into solid are much larger than the rejected fluxes. The interatomic interactions in liquid and solid do not affect the liquid–solid distribution [39,40]. On the other hand, a large $\;\Gamma_{l}$ corresponds to low supersaturations of liquid, where thermodynamics starts to play an important role. The limiting behavior described by the equilibrium liquid–solid distribution at the no-growth conditions [40,42,43,44] is given by:

(4) $$y_{eq}=\frac{x}{x+\beta_{l}(1-x)e^{\omega (2x-1)}\;}.$$

The kinetic distribution given by Equation (3) contains no thermodynamic parameters of group V atoms in liquid, because their diffusion transport depends only on the group III fraction in a droplet (in the Au-catalyzed VLS growth). Conversely, the equilibrium distribution given by Equation (4) is fully determined by thermodynamics and contains no kinetic parameters.

The interplay of kinetic and thermodynamic factors influencing the liquid–solid distribution is determined by the magnitude of $\Gamma_{l}$, which contains the product ${\chi_{tot}\chi }_{C}$ in the denominator. While the concentration of group III atoms in liquid is very close to unity in the self-catalyzed growth and can easily be measured after growth [27] (or even during growth using in situ monitoring inside a transmission electron microscope (TEM) [37]), nothing can be said about $\chi_{tot}$. This makes Equation (2) alone rather useless for the compositional modeling. It is clear that the value of $\chi_{tot}$ is determined by the vapor fluxes. In Ref. [40], we have shown that the uncertainty in the unknown concentrations of group V atoms in liquid can be fully circumvented in the steady-state VLS growth regime. At a constant droplet volume, the total influx of group V atoms A and B minus their desorption fluxes equals the influx of group III C atoms (in the absence of their desorption or downward diffusion fluxes from the droplet onto the NW sidewalls, with subsequent evaporation [11]). The influxes of group V atoms [39,40,53] include the direct impingement, re-emission [39,40,53] and possibly surface diffusion of group V adatoms from the NW sidewalls over a short distance [54,55,56]. In this work, we neglect the surface diffusion of group V adatoms [39,40,53] and assume their 100% adsorption at the droplet surface. In this case, the result of Ref. [40] is reduced to

(5) $$\chi_{tot}^{2}=\frac{\varepsilon -1}{K\left[y^{2}+\zeta (1-{y)}^{2}\right]},$$

(6) $$z=\frac{x}{\varepsilon }+\left[1-\frac{1}{\varepsilon }\right]\frac{1}{1+\zeta {\left[\left(1-y\right)/y\right]}^{2}},$$

with the parameters

(7) $$\varepsilon =\frac{2\sigma_{5}(I_{A_{2}}+I_{B_{2}})}{\sigma_{C}I_{C}},\;\zeta =\frac{I_{B_{2}}^{0}}{I_{A_{2}}^{0}}e^{2(\psi_{B}-\psi_{A})},\;K=\frac{2\sigma_{5}I_{A_{2}}^{0}e^{2\psi_{A}}}{\sigma_{C}I_{C}}.$$

Here, $I_{A_{2}}$ and $I_{B_{2}}$ are the vapor fluxes of the group V dimers $A_{2}$ and *B*_2_, with

(8) $$z=\frac{I_{A_{2}}}{I_{A_{2}}+I_{B_{2}}}$$

as the fraction of A atoms in vapor, and $\sigma_{5}=\sigma_{A}=\sigma_{B}$ as the geometrical coefficient which depends on the droplet contact angle. $I_{C}$ denotes the vapor flux of group III C atoms. The term $\sigma_{C}I_{C}$ gives their total flux into the droplet including surface diffusion. Therefore, ε is the effective total V/III flux ratio for the fluxes entering the droplet, which is different from the V/III ratio in vapor. $I_{A_{2}}^{0}$ and $I_{B_{2}}^{0}$ are defined as the vapor fluxes of A_2_ and B_2_ dimers at equilibrium with pure A and B liquids [40]. The desorption fluxes of elements k = A, B are given by $I_{k_{2}}^{0}\exp\left(2\psi_{k}\right)\chi_{k}^{2}$, because group V elements desorb in the form of dimers [39,40,51,52,53]. Hence, ζ gives the ratio of the pre-factors in the desorption rates for dimers B_2_ over A_2_ from a Au-III liquid. Finally, K is the ratio of the pre-factor in the desorption flux of A_2_ dimers over the total influx of group III atoms.

Equation (5) gives the dependence of the total concentration of group V atoms in the droplet on the effective V/III flux ratio ε and the liquid composition y. The vapor–solid distribution in Equation (6) is a combination of the linear function z=x and the desorption-related term. The latter depends on the liquid–solid distribution y(x). The weights of these competing terms are regulated by the effective total V/III flux ratio ε. For example, at ε→1 group V atoms are not allowed to desorb from the droplet in the steady-state VLS growth regime. Then, $\chi_{tot}$ tends to zero according to Equation (5). The liquid state in this regime has no influence on the vapor–solid distribution, which simply equals z=x [39,40,41]. In the general case, the parameter $\Gamma_{l}$ in Equation (2) for $y\left(x\right)\;$ becomes a function of y and ε according to Equation (5). Inferring the explicit dependence $y\left(x\right)$ from Equation (2) now requires the solution of a quadratic equation for y, which is obtained in the form

(9) $$y=y_{kin}G,\;\;G=\frac{1-\zeta F^{2}/y_{kin}+F\sqrt{1+u{\left(1-x\right)}^{2}/x^{2}-\zeta {\left(F/y_{kin}\right)}^{2}}}{1-(1+\zeta )F^{2}}.$$

Here,$\;y_{kin}(x)$ is the LM liquid–solid distribution given by Equation (3). F(x) is a complex function of x that contains many parameters of the liquid phase and the interaction constant ω:

(10) $$F\left(x\right)=\Gamma_{*}\frac{x(1-x)}{x+c_{l}(1-x)}\left[c_{l}e^{\omega {\left(1-x\right)}^{2}}-\beta_{l}e^{\omega x^{2}}\right].$$

The important parameter of our model,

(11) $$\Gamma_{*}=\frac{1}{\sqrt{\varepsilon -1}}\sqrt{\frac{2\sigma_{5}I_{A_{2}}^{0}}{\sigma_{C}I_{C}}}\frac{e^{-∆\mu_{AC}^{0}-\psi_{C}}}{\chi_{C}},$$

is independent of the concentrations of group V atoms A and B in liquid, because the interactions with negligible fractions of these atoms in $\psi_{C}$ can be safely neglected [43] (with a possible exception of Sb). The parameter $\Gamma_{*}$ is inversely proportional to the supersaturation of vapor with respect to the solid. It contains the vapor fluxes, the V/III flux ratio and the activity of group III atoms in liquid $\chi_{C}\exp\left(\psi_{C}\right)$, which is very close to unity in the self-catalyzed VLS process and depends only on $\chi_{C}$ in the Au-catalyzed VLS process. Another important parameter,

(12) $$u=\zeta c_{l}^{2}=\frac{I_{B_{2}}^{0}}{I_{A_{2}}^{0}}{\left(\frac{D_{A}}{D_{B}}\right)}^{2},$$

lumps together the ratio of the pre-factors in the desorption rates of B_2_ over A_2_ dimers and the ratio of the diffusion coefficients of A over B atoms in pure group V liquids. It is also independent of the liquid composition, but highly sensitive to temperature. The u value is larger for A_x_B_1−x_C materials with larger desorption and lower diffusivities of B species with respect to A. The function G in Equation (9) describes the thermodynamic factors in the liquid–solid distribution. In the regimes where the depletion of a catalyst droplet with its group V atoms with the ML progression (where only our model applies) is negligible, the thermodynamic function $F\left(x\right)$ must be small, which requires $\Gamma_{*}\ll 1$. Neglecting all terms containing ${\;F}^{2}$ in Equation (9), we obtain the linearized liquid–solid distribution

(13) $$y≅y_{kin}\left[1+\sqrt{1+u{\left(\frac{1-x}{x}\right)}^{2}}F\right].$$

In the limit $\Gamma_{*}\to 0$, corresponding to high levels of supersaturation due to large material inputs from vapor, we simply have G=1, and the liquid–solid distribution becomes purely kinetic. At $y=y_{kin}$, the vapor–solid distribution given by Equation (6) is reduced to

(14) $$z=\frac{x}{\varepsilon }+\left[1-\frac{1}{\varepsilon }\right]\frac{x^{2}}{x^{2}+u{\left(1-x\right)}^{2}}.$$

This distribution is a combination of the linear kinetic and non-linear desorption-related terms. The latter contains the single parameter u given by Equation (12). The pre-factors in the desorption rates $I_{k_{2}}^{0}$ are well-known for any group V dimers [53] and will be analyzed in the next section. The ratio $D_{A}/D_{B}$ is generally unknown, but can be deduced from fitting the compositional data. It is interesting to note that the parameter $\Gamma_{*}$ decreases with ε according to Equation (11). At low ε approaching unity, the liquid composition has no influence on the vapor–solid distribution according to Equation (6). Therefore, Equation (14) should be applicable in a wide range of growth conditions. This model fully circumvents the uncertainty in the unknown concentrations of group V atoms A and B in liquid in the stationary vapor–solid distribution. Furthermore, no dependence on the liquid composition is left in Equation (14). The validity of this model depends on the technologically controlled parameters such as the effective total V/III ratio ε and temperature, which largely influences the desorption fluxes. In the general case, however, the vapor–solid distribution given by Equation (6) depends on the liquid state because it contains the liquid–solid distribution given by Equations (9) or (13). This will be considered in the next section for different VLS IIIV_x_V_1−x_ NWs.

## 3. Results and Discussion

**Figure 1:**
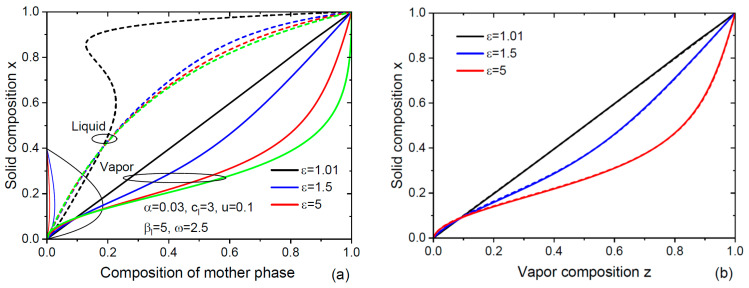
(**a**) Liquid–solid (bold dashed lines) and vapor–solid (bold solid lines) distributions for a model VLS IIIV_x_V_1−x_ system at a fixed temperature and different effective V/III ratios of ε  from 1 to infinity. As ε increases, the liquid–solid distribution transitions from a curved shape containing the miscibility gap region at a low ε=1.01 to the LM shape $y_{kin}\;$at a high ε (green solid and dashed lines). The vapor–solid distribution at ε=1.01 is given by x=z and has nothing to do with the non-linear shape of the liquid–solid distribution. No material segregation actually occurs in NWs. As ε increases, the vapor–solid distribution transitions to a non-linear shape given by Equation (14) at ε→0, which is entirely determined by the parameter u. The functions F(x) for each ε are shown by solid lines, and remain much smaller than unity in all cases. (**b**) Vapor–solid distributions obtained from the general expression given by Equation (6) (solid lines) and its approximation given by Equation (14) (dashed lines). The curves are indistinguishable, showing that Equation (14) works perfectly well for any V/III flux ratio in this example.

Figure 1a shows the liquid–solid and vapor–solid distributions of VLS IIIV_x_V_1−x_ NWs grown under variable V/III flux ratios corresponding to ε= 1.01, 1.5 and 5. Calculations are performed for a model system with a fixed $c_{l}=3$, u=0.1, $\beta_{l}=5$, ω=2.5 and $\Gamma_{*}=\alpha /\sqrt{\varepsilon -1}$ with α=0.03. These fixed parameters correspond to a given growth temperature and percentage of Au in a catalyst droplet. The curves are obtained from Equation (13) for y(x) and from (6) for z(x), with F(x) given by Equation (10). The liquid–solid distribution x(y) at a low ε of 1.01 contains the miscibility gap region as ω>2. However, this does not affect the linear vapor–solid distribution z=x, which occurs due to the negligible desorption of both group V atoms. The miscibility gap describes the forbidden states in liquid rather than in solid. As ε increases, the liquid–solid distribution rapidly acquires the LM kinetic shape given by Equation (3). Simultaneously, the vapor–solid distribution transitions to the non-linear curve given by Equation (14) at ε→1. Figure 1b shows a comparison of the general vapor–solid distribution given by Equation (6) (which contains y(x)) and its approximate form given by Equation (14) (which is independent of the liquid composition and interactions in solid). The curves are indistinguishable, showing that Equation (14) provides excellent approximation for any ε with these model parameters. It was previously noted that the liquid–solid distribution is almost useless for control over the stationary NW composition [39,40,46] (however, the dependence of y(x) becomes very important in modeling the interfacial abruptness in axial NW heterostructures [38,42,43]). This property is well illustrated in Figure 1. The vapor–solid distributions shown in the figure qualitatively describe the transition from the linear curve z=x to a non-linear shape with the suppressed incorporation of Sb with the total V/III flux ratio, observed in Au-catalyzed InSb_x_As_1−x_ NWs [12]. A quantitative analysis of these data based on Equation (14) will be presented elsewhere.

Let us now discuss the temperature-dependent desorption pre-factors $I_{k_{2}}^{0}$ and their ratio, which enter our control parameters $\Gamma_{*}$ and u. We defined the fluxes $I_{k_{2}}^{0}$ as the vapor fluxes of the A_2_ and B_2_ dimers that are at equilibrium with pure liquids A and B. However, practical calculations of the desorption fluxes for each group V species using the thermodynamic data of Refs. [57,58] are more easily performed by choosing the vapor of dimers k_2_ at total pressure P and temperature T as the reference state [53]. In this case, we introduce the reference chemical potentials of the perfect gas of k_2_ dimers at pressure P:

(15) $$\mu_{k_{2}}^{g,ref}=2\mu_{A}^{l,0}+ln\left(\frac{P}{P_{k_{2}}^{0}}\right).$$

The characteristic pressure $\;P_{k_{2}}^{0}$ is related to the flux $I_{k_{2}}^{0}$ according to [53].

(16) $$I_{k_{2}}^{0}=\frac{P_{k_{2}}^{0}}{\sqrt{2\pi m_{k_{2}}k_{B}T}}.$$

Here, $\;m_{k_{2}}=2m_{k}$ is the mass of dimer k_2_ and $m_{k}$ is the atomic mass. Using Equations (15) and (16), we obtain

(17) $$P_{k_{2}}^{0}=Pe^{2\mu_{k}^{l,0}-\mu_{k_{2}}^{g,ref}},$$

and

(18) $$\frac{I_{B_{2}}^{0}}{I_{A_{2}}^{0}}=\sqrt{\frac{m_{A}}{m_{B}}}e^{2\left(\mu_{B}^{l,0}-\mu_{A}^{l,0}\right)+\mu_{A_{2}}^{g,ref}-\mu_{B_{2}}^{g,ref}}.$$

The desorption pre-factors $I_{k_{2}}^{0}\;$ and their ratios can now be calculated as functions of temperature for As_2_, P_2_ and Sb_2_ species using the data of Refs. [57,58], with P= 10^5^ Pa. The desorption fluxes in Equation (16) are in nm^−2^ s^−1^. Expressing them in ML/s requires multiplication by the factor $\Omega_{s}/h$, with $\Omega_{s}$ as the elementary volume per III–V pair in solid and h as the height of an ML.

**Figure 2:**
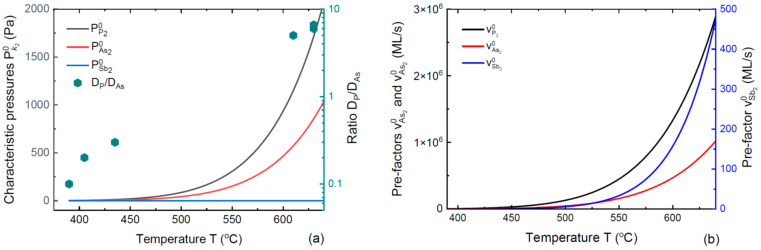
(**a**) Temperature-dependent characteristic pressures and (**b**) pre-factors in the desorption rates in ML/s for dimers P_2_, As_2_ and Sb_2_, calculated using Equations (16) and (17) and the data of Refs. [57,58]. Symbols in panel (a) show the ratios of the diffusion coefficients $D_{P}/D_{As}$ for atoms P and As in pure group V liquids, obtained from the fits to the compositional data in Figure 4.

Figure 2 shows the characteristic pressures $P_{k_{2}}^{0}$ and the corresponding desorption pre-factors $v_{k_{2}}^{0}=(\Omega_{s}/h)I_{k_{2}}^{0}$ in ML/s for dimers P_2_, As_2_ and Sb_2_. The curves are obtained from Equations (16) and (17) using the data of Refs. [57,58]. Calculations for As_2_ dimers are the same as in Ref. [53], and reproduce the desorption flux of As_2_ $I_{{As}_{2}}^{0}\exp\left(2\psi_{As}\right)\chi_{As}^{2}$ as a function of temperature and As concentration given in this work. The flux $v_{k_{2}}^{0}$ is highest for P_2_. The ratio $I_{{As}_{2}}^{0}/I_{P_{2}}^{0}≅0.31$ is almost constant over the entire temperature range shown in the figure. The characteristic pressure $P_{{Sb}_{2}}^{0}$ and the corresponding flux$\;v_{{Sb}_{2}}^{0}$ are three orders of magnitude lower. Hence, Sb_2_ dimers desorb much less than As_2_ and P_2_. The actual desorption rates $I_{k_{2}}^{0}\exp\left(2\psi_{k}\right)\chi_{k}^{2}$ for k = P and As appear quite high, for example, in the order of 1 ML/s for self-catalyzed GaAs NWs at 610 °C and $\chi_{As}=0.02$. This explains the importance of re-emitted group V species [53,59] to enable NW growth by molecular beam epitaxy (MBE) with modest inputs of group V atoms (~1 ML/s). The re-emission of As species yields a multiplying factor of ~4 for the droplet flux according to Ref. [53].

**Figure 3:**
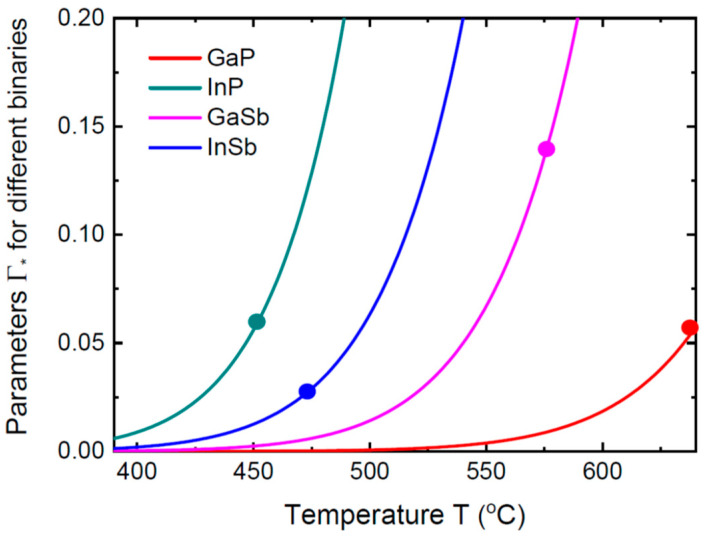
Temperature-dependent parameters $\Gamma_{*}$ for GaP, InP, GaSb and InSb binaries. Circles correspond to the maximum temperatures for the typical VLS growths of GaP_x_As_1−x_, InP_x_As_1−x_, GaSb_x_As_1−x_ and InSb_x_As_1−x_ NWs. For GaP_x_As_1−x_, InP_x_As_1−x_ and InSb_x_As_1−x_ systems, the values of $\Gamma_{*}$ remain smaller than ~0.05 in the interesting temperature windows, which fully justifies the independence of the stationary NW compositions on the liquid state. For the GaSb_x_As_1−x_ system, the parameter $\Gamma_{*}$ is negligibly small for temperatures below 550 °C, but increases to more than 0.2 at 590 °C. Further increases in temperature lead to the liquid-dependent compositional trends.

These calculations allow us to deduce the typical values of the parameter $\Gamma_{*}$ which determines the importance of the liquid composition in the stationary vapor–solid distributions. Figure 3 shows the temperature-dependent $\Gamma_{*}$ for different material combinations. The curves are obtained from Equation (11), assuming the self-catalyzed VLS process or group III-rich droplets in the Au-catalyzed VLS process ($\psi_{C}≅0,\;\chi_{C}≅1$), ε=2, $2\sigma_{5}/\sigma_{C}=0.2$ to account for the surface diffusion of group III adatoms, and an $I_{c}$ equivalent to 1 ML/s. The temperature-dependent $∆\mu_{AC}^{0}$ is obtained using the data of Refs. [57,58]. These calculations apply to VLS GaP_x_As_1−x_, InP_x_As_1−x_, GaSb_x_As_1−x_ and InSb_x_As_1−x_ NWs, that is, when we choose P and Sb as the element A in the ternary A_x_B_1−x_C alloy. It is seen that the values of $\Gamma_{*}$ remain negligibly small (less than 0.07) for the typical VLS growth temperatures of GaPAs (below 640 °C [19,20,21,29,30,31], InPAs (below 450 °C [17,27]), InSbAs (below 470 °C [12,13]) and GaSbAs (below 550 °C [16,60]) NWs. Therefore, our model based on Equation (14) is fully justified in these temperature windows. Increasing the growth temperature for GaSbAs NWs up to 590 °C [60], where the value of $\Gamma_{*}$ increases above 0.2, may lead to a liquid-dependent compositional trend. Overall, this example shows that the stationary vapor-solid distributions of most VLS IIIV_x_V_1−x_ NWs should be almost independent of the liquid composition in many practical cases, and primarily determined by the vapor fluxes, diffusion transport rates in pure liquids and desorption. In other words, the composition of VLS IIIV_x_V_1−x_ NWs is controlled by (i) the effective total V/III flux ratio ε and (ii) the growth temperature. This is described by our Equation (14), where the temperature-dependent functions are lumped together in the single parameter u. However, the validity of this simplified model should be carefully checked for each VLS ternary system at a given temperature and V/III flux ratio in vapor, with the known group III content in the droplet Au-catalyzed NWs.

**Figure 4:**
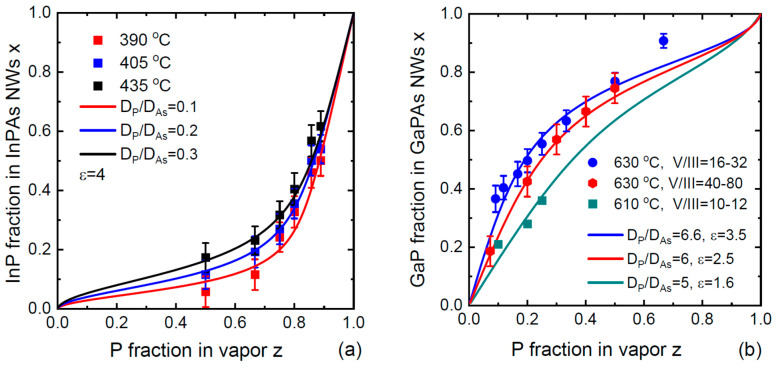
Stationary vapor–solid distributions of (**a**) Au-catalyzed InP_x_As_1−x_ NW sections at three different temperatures shown in the legend [17], and (**b**) Ga-catalyzed GaP_x_As_1−x_ NWs/NW sections at different temperatures and total V/flux ratios shown in the legend [19,21,30] (symbols), fitted by Equation (14) with the parameters shown in the legends (lines).

Figure 4a shows the compositional data for Au-catalyzed InP_x_As_1−x_ NWs obtained by Persson and coauthors in Ref. [17]. These NWs were grown by chemical beam epitaxy on InAs(111)B substrates using 50 nm diameter colloidal Au droplets, which resulted in ~60 nm diameter NWs. The growth started with InAs NW stems and continued with InP_x_As_1−x_ top sections grown at 390 °C, 405 °C and 435 °C. The total V/III flux ratio in vapor during the growth of InPAs sections was in the range of 30 to 45. Figure 4b shows the compositional data for Ga-catalyzed GaP_x_As_1−x_ NWs of Refs. [19,21,30]. GaP_x_As_1−x_ NWs [19,21] or GaP_x_As_1−x_ sections in GaP NWs [30] were grown by MBE on Si(111) substrates. Himwas and coauthors [19] grew the NWs at 610 °C under the total V/III flux ratios in vapor ranging from 10 to 12. Zhang and coauthors [21] and Bolshakov and coauthors [30] grew the NWs at 630 °C under higher total V/III ratios in vapor, ranging from 40 to 80 in Ref. [21] and from 16 to 32 in Ref. [31]. According to Figure 3, the values of $\Gamma_{*}$ for both material systems at the growth temperatures are negligibly small and Equation (14) should be directly applicable.

It is seen that the simplified model indeed provides excellent fits to all the data, apart from one data point corresponding to the maximum fraction of GaP in GaP_x_As_1−x_ NW sections in Figure 4b. The values of ε in all cases are significantly lower than the total V/III ratios in vapor. This is explained by the surface diffusion of group III adatoms from different surfaces [11] and was previously discussed in Refs. [12,38,39] in connection with the vapor–solid distributions of VLS IIIV_x_V_1−x_ NWs. In the VLS growth process, a catalyst droplet serves as an efficient material collector for group III species, changing the material balance at the NW top. As a result, the effective V/III flux ratio entering the droplet is largely reduced with respect to the total V/III ratio in vapor. In our model, this corresponds to $2\sigma_{5}/\sigma_{C}<1$ or even $2\sigma_{5}/\sigma_{C}\ll 1$. This ratio depends on many factors including the NW height, surface density, temperature-dependent diffusion lengths of group III adatoms on the NW sidewalls and/or substrate surface and the re-emission of group V and III atoms from the substrate. The fitting values of ε range from 1.6 to 4, which leads to the non-linearity of the vapor–solid distributions arising from the second temperature-dependent term in our Equation (14). This term contains the fitting parameter u. From Figure 2, the ratio $I_{{As}_{2}}^{0}/I_{P_{2}}^{0}$ in Equation (12) for u is estimated at ≅0.31 for the entire temperature range from 390 °C to 640 °C. The ratio of diffusion coefficients $D_{P}/D_{As}$ for atoms P and As in pure P and As liquids is unknown. From the fits shown in Figure 4, we deduce the values of ${\;D}_{P}/D_{As}$ shown in Figure 2a at different temperatures. This ratio is around 0.1 at the lowest temperature of 390 °C, and gradually increases with temperature up to ~7 at 630 °C. This behavior calls for a discussion and requires further studies. It should be noted that the uncertainty in the diffusion transport coefficients of group V and group III atoms through liquid is present in all kinetic models for NW composition [38,39,40,45]. In our model, we eliminated any possible influence of binary interactions in liquid on the diffusion transport. However, this formal approach reduces the diffusion coefficients to the unknown values related to pure group V liquids. This is not essential, and we may instead consider the diffusion coefficients ${\;D}_{k}exp(\psi_{k})$ in Au-group III or pure group III liquids and their ratio. In any case, in-depth analysis of the diffusion transport of different atoms in liquid catalysts is urgently required, because different diffusivities of atoms A and B affect the composition of III–V ternary NWs grown by the VLS method.

To summarize our findings, the general vapor–solid distribution of VLS IIIV_x_V_1−x_ NWs $z\left(x\right)\;$ (Equation (6) with the liquid–solid distribution y(x) given by Equation (9)) contains the characteristics of liquid and interactions in solid. However, our calculations show that the control parameter $\Gamma_{*}$ is small under the typical growth conditions (temperatures, total fluxes and V/III flux ratios) for VLS IIIV_x_V_1−x_ NWs. In this case, our solution is reduced to the two-parameter Equation (14) which contains no interactions in liquid or solid. Table 1 summarizes the control parameters of the vapor–solid distribution, their role in the NW composition and dependence on the technologically controlled growth parameters. These trends can be used directly for the compositional control over VLS IIIV_x_V_1−x_ NWs by the growth parameter tuning.

## 4. Conclusions and Outlook

The main result of this study is the general vapor–solid distribution of VLS IIIV_x_V_1−x_ NWs given by Equation (6) with y(x) given by Equation (9), and its simplified form given by Equation (14). The interplay of kinetic and thermodynamic factors influencing the compositional trends in IIIV_x_V_1−x_ NWs is summarized in Table 1. We have shown that the importance of the liquid composition and binary interactions in solids in the vapor–solid distribution is regulated by the parameter $\Gamma_{*}$. It appears negligibly small in the VLS growth regimes without the depletion of a catalyst droplet with its group V atoms with the ML progression. The realization of this regime requires large enough droplets and high enough concentrations of group V atoms at nucleation [49,51,52]. In this case, the stationary vapor–solid distribution is independent of the liquid composition and given by a combination of the linear function z=x and a non-linear temperature-dependent term with the parameter u. The weights of the two terms are regulated by the effective V/III flux ratio ε. The parameter u is a product of the known ratio of the characteristic desorption rates of B_2_ over A_2_ dimers and the squared ratio of the unknown diffusion coefficients ${\left(D_{A}/D_{B}\right)}^{2}$. The model fits very well the compositional data on VLS InPAs and GaPAs NWs, and should be applicable to all VLS IIIV_x_V_1−x_ NWs. Most importantly, we have demonstrated that the stationary composition of VLS IIIV_x_V_1−x_ NWs in a wide range of conditions is determined only by temperature and the V/III flux ratio. These findings should be useful for compositional control and bandgap engineering in VLS NWs for electronic and optoelectronic applications, and can be extended to other material systems and hybrid NWs [61,62,63].

The liquid–solid distribution, which is only required for the time-dependent modeling of NW heterointerfaces [42,43], is given by the LM formula with the parameter $c_{l}$. This parameter also contains the unknown ratio $D_{A}/D_{B}$. Therefore, further studies should consider in detail the diffusion of group V atoms in a liquid catalyst. A decrease in supersaturation in liquid and its possible drop to zero, resulting in a stopping size after which the ML progresses slowly at the rate of refill from vapor, was considered theoretically and verified experimentally for wurtzite GaAs NWs grown by Ga-catalyzed MBE inside a TEM [49,50,51,52]. This interesting effect was never considered for VLS III–V ternary NWs. We plan to study this growth regime and its influence on the NW composition in a future work.

## Figures and Tables

**Table 1:** Summary of the control parameters in vapor–solid distribution of VLS IIIV_x_V_1−x_ NWs.

Parameter	Role in NW Composition	Temperature Dependence	Group III Flux Dependence	V/III Ratio Dependence
Vapor-related supersaturation parameter $\Gamma_{*}$	Eliminates interactions in liquid and solid at $\Gamma_{*}\ll 1$	Increases with T	Decreases with $I_{C}$	Decreases with V/III flux ratio in vapor
Effective V/III flux ratio for the droplet ε	Eliminates non-linearity of the vapor–solid distribution at ε→1	Contains temperature-dependent diffusion length of group III adatoms	Independent	Increases with V/III flux ratio in vapor
Desorption/transport parameter u	Leads to non-linear vapor–solid distribution at large ε	Contains unknown ratio of diffusion coefficients in liquid. Increases with T for IIIP_x_As_1−x_ NWs according to our results	Independent	Independent

## Data Availability

Data are contained within the article.
